# Management of Retrograde Peri-Implantitis Using an Air-Abrasive Device, Er,Cr:YSGG Laser, and Guided Bone Regeneration

**DOI:** 10.1155/2018/7283240

**Published:** 2018-04-15

**Authors:** Nikolaos Soldatos, Georgios E. Romanos, Michelle Michaiel, Ali Sajadi, Nikola Angelov, Robin Weltman

**Affiliations:** ^1^Department of Periodontics and Dental Hygiene, School of Dentistry, University of Texas Health Science Center at Houston, Houston, TX, USA; ^2^Department of Periodontology, School of Dental Medicine, Stony Brook University, Stony Brook, NY, USA; ^3^Department of Oral Surgery and Implant Dentistry, Johann Wolfgang Goethe University of Frankfurt, Frankfurt, Germany

## Abstract

**Background:**

The placement of an implant in a previously infected site is an important etiologic factor contributing to implant failure. The aim of this case report is to present the management of retrograde peri-implantitis (RPI) in a first maxillary molar site, 2 years after the implant placement. The RPI was treated using an air-abrasive device, Er,Cr:YSGG laser, and guided bone regeneration (GBR).

**Case Description:**

A 65-year-old Caucasian male presented with a draining fistula associated with an implant at tooth #3. Tooth #3 revealed periapical radiolucency two years before the implant placement. Tooth #3 was extracted, and a ridge preservation procedure was performed followed by implant rehabilitation. A periapical radiograph (PA) showed lack of bone density around the implant apex. The site was decontaminated with an air-abrasive device and Er,Cr:YSGG laser, and GBR was performed. The patient was seen every two weeks until suture removal, followed by monthly visits for 12 months. The periapical X-rays, from 6 to 13 months postoperatively, showed increased bone density around the implant apex, with no signs of residual clinical or radiographic pathology and probing depths ≤4 mm.

**Conclusions:**

The etiology of RPI in this case was the placement of an implant in a previously infected site. The use of an air-abrasive device, Er,Cr:YSGG, and GBR was utilized to treat this case of RPI. The site was monitored for 13 months, and increased radiographic bone density was noted.

## 1. Introduction

Retrograde peri-implantitis (RPI) is termed as a symptomatic periapical lesion, developed after implant placement, while the coronal portion of the implant remains fully osseointegrated [1]. It was initially described in 1992 by McAllister et al. where they described two cases of RPI caused by bacteria remained in the extraction socket [2]. In 1993, Sussman and Moss defined it as “localized osteomyelitis secondary due to endodontic pathology” [3]. In 1995, Reiser and Nevins described it as “active implant periapical lesion” [4]. Piattelli et al., in 1998, histologically examined an implant that was removed due to periapical radiolucency. They discovered the presence of necrotic bone inside the antirotational hole and the demineralization of the bordered trabecular bone [5]. Esposito et al. in 1998 considered the placement of an implant in a previously infected site to be an important factor contributing to implant failure [6].

Etiological factors of RPI are divided to those which occur at the time of implant placement and those due to a preexisting disease (Table 1) [7–9]. Moreover, an HIV-related infection was described as an etiological factor for RPI as well [7].

Bacteria can be encapsulated in edentulous areas, up to 1 year after the extraction [10]. Therefore, many implants that developed RPI, where previously root canal treated teeth, were present [10]. The reported prevalence of RPI is very low (0.26%), but it can be increased up to 7.8% when there is a history of a root canal treatment of an adjacent tooth, next to the implant site [11]. Studies support the launch of symptoms from 1 week after implant placement, up to 4 years later [12–15]. The symptoms vary from presence of a fistula tract to pain and swelling. The presence of a fistula tract has shown the highest prevalence (65.6%) [9, 12, 15, 16]. Maxillary implant sites (78%) seem to be more exposed to RPI compared to mandibular (18%) [12, 15, 16]. Reiser and Nevins connected that finding to the higher frequency of radicular cysts in the maxilla [4]. Bhaskar connected the higher frequency of maxillary radicular cysts with the epithelial rests of Malassez, which seem to be more numerous in the maxilla than in the mandible [17].

The aim of this case report was to present the management of RPI in a first maxillary molar site, 2 years after the implant placement. The implant apex was located in close proximity to where the periapical radiolucency of tooth #3 was in 2013. The diagnosis was RPI, and the affected implant was treated successfully using the combination of an air-abrasive device, Er,Cr:YSGG laser, and guided bone regeneration (GBR).

## 2. Case Presentation

A 65-year-old Caucasian male, nonsmoker, ASA II with a significant medical history for hypertension, was referred in 2016 to the Graduate Periodontics Clinic at the University of Texas Health Science Center at Houston. His chief complaint was “*they said I have an abscess around my implant*.” The patient had been prescribed clindamycin 300 mg for a week by his referring predoctoral student of the same University. Clinically, a draining fistula was present at the tooth #3 implant site, just apical to the mucogingival junction, measuring approximately 3 × 3 mm. Comprehensive periodontal and radiographic evaluations were performed. The patient was very meticulous with oral hygiene, and there was absence in bleeding on probing and mobility, with thick gingival biotype. The periodontal pocket measurements around the implant were ≤4 mm. The diagnosis associated with the implant at the area of tooth #3 was RPI.

**Figure 1:**
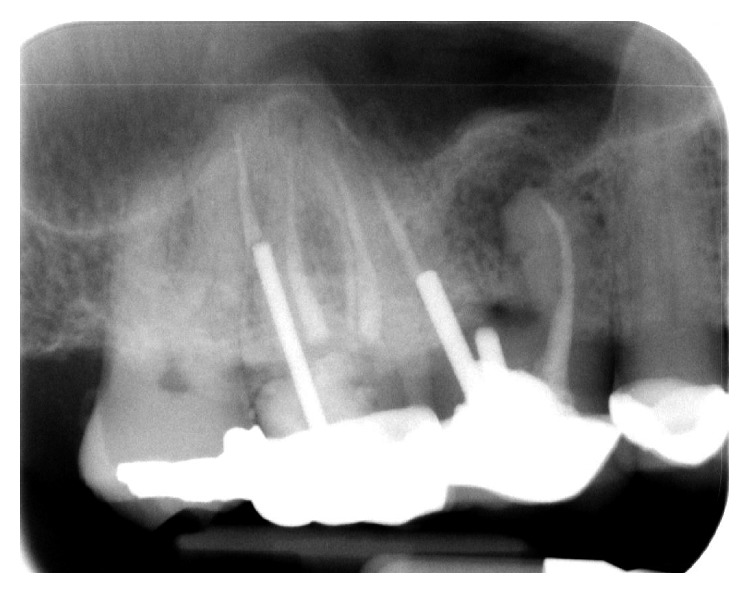
Radiograph of tooth #3 showed periapical radiolucency on the mesial buccal root.

**Figure 2:**
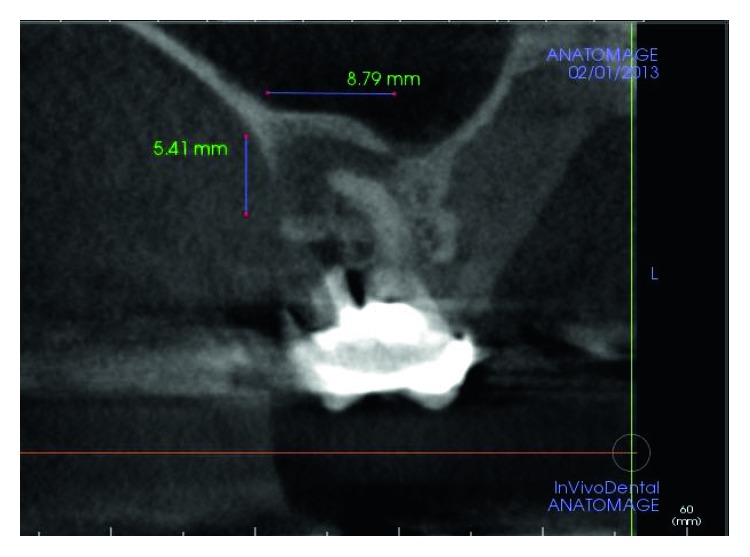
A cone beam CT of tooth #3 in 2013 showed periapical radiolucency of the mesial buccal root (∼5.4 × 8.8 mm).

**Figure 3:**
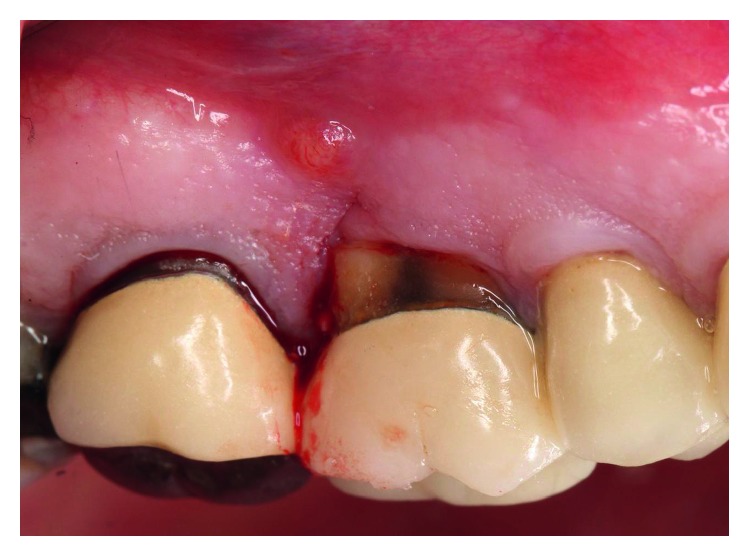
Intraoral picture of tooth #3 at the time of tooth extraction. A draining fistula was noted on the distal surface of #3.

**Figure 4:**
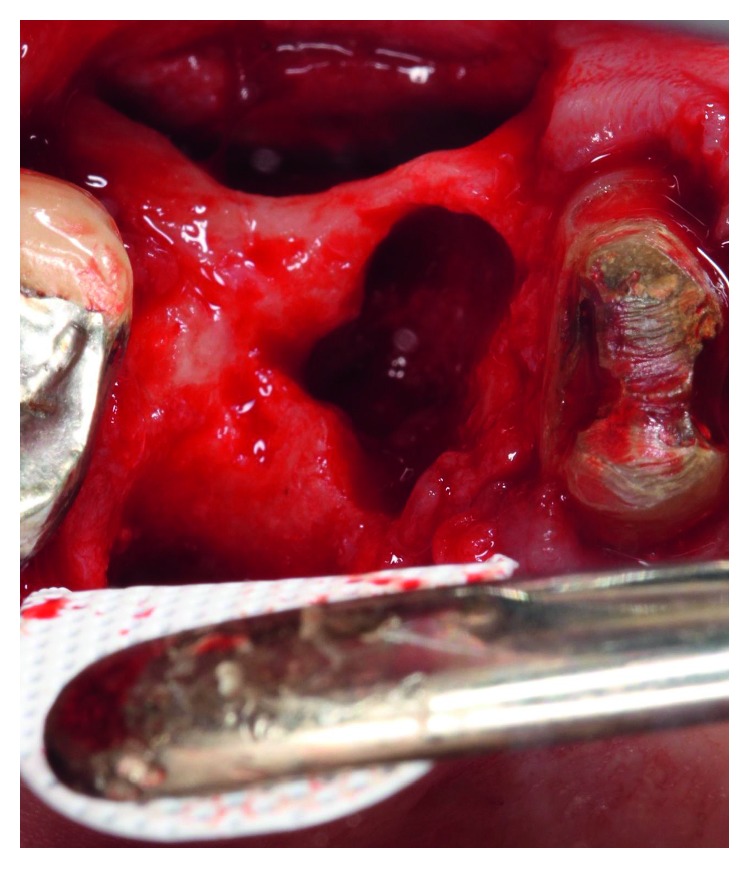
Intraoral picture of the extraction site of #3. The buccal plate showed horizontal deficiency. The nonresorbable high density PTFE membrane was placed on the lingual aspect of #3.

**Figure 5:**
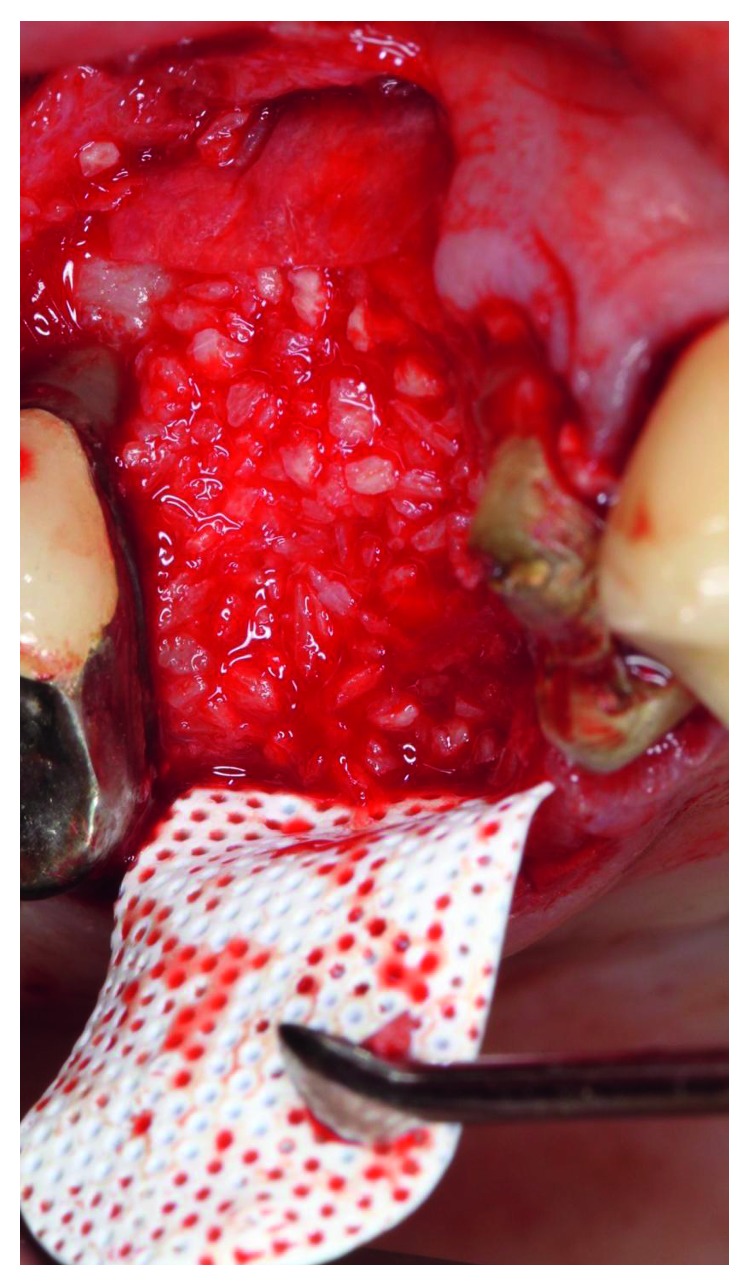
The collagen membrane was placed on the buccal aspect to cover the grafted buccal plate. The nonresorbable high density PTFE membrane was placed on the lingual aspect to cover the grafted socket.

**Figure 6:**
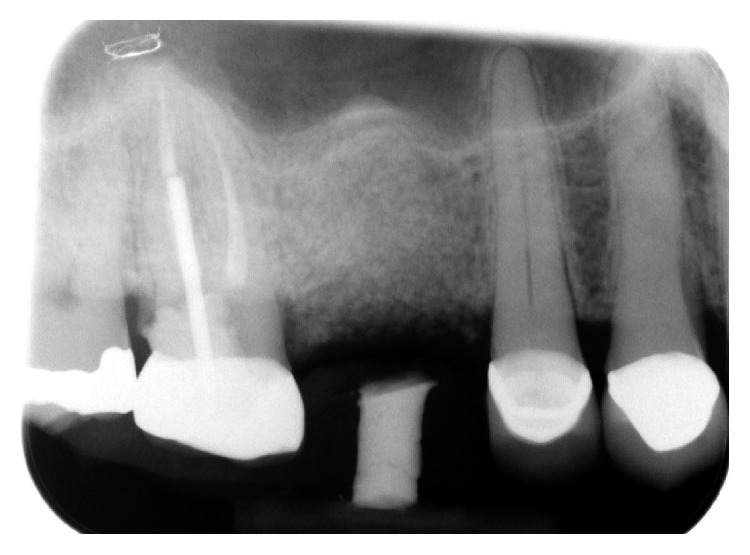
A periapical radiograph 3 months after the extraction and ridge preservation showed no signs of residual pathology.

**Figure 7:**
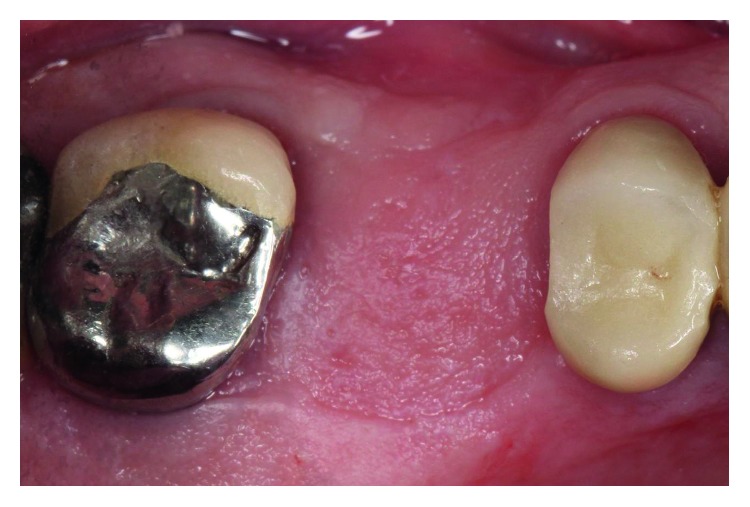
The site of #3, 3 months after the ridge preservation showed complete epithelization.

**Figure 8:**
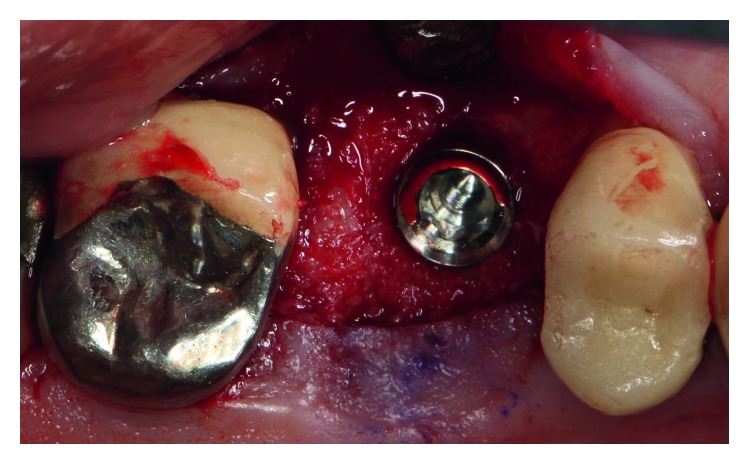
A 4.7/11 mm endosseous implant was placed at site #3 with the use of osteotome sinus floor elevation technique.

**Figure 9:**
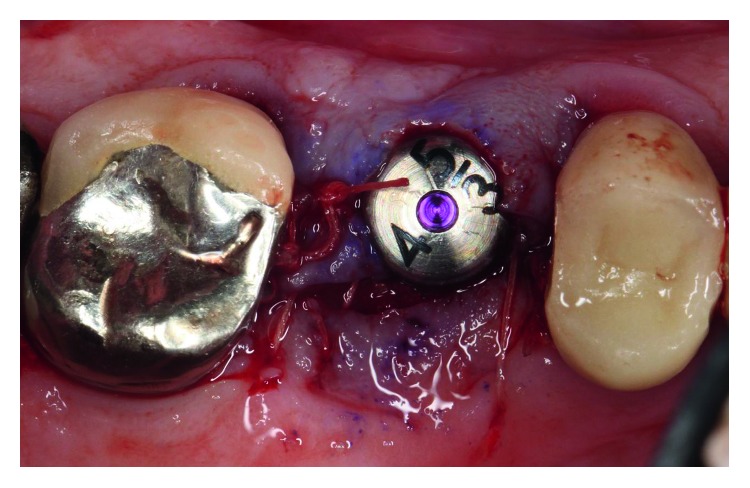
The implant was torqued in 35 N/cm, and a healing abutment was placed. The site was sutured with three 5–0 chromic gut single-interrupted sutures.

**Figure 10:**
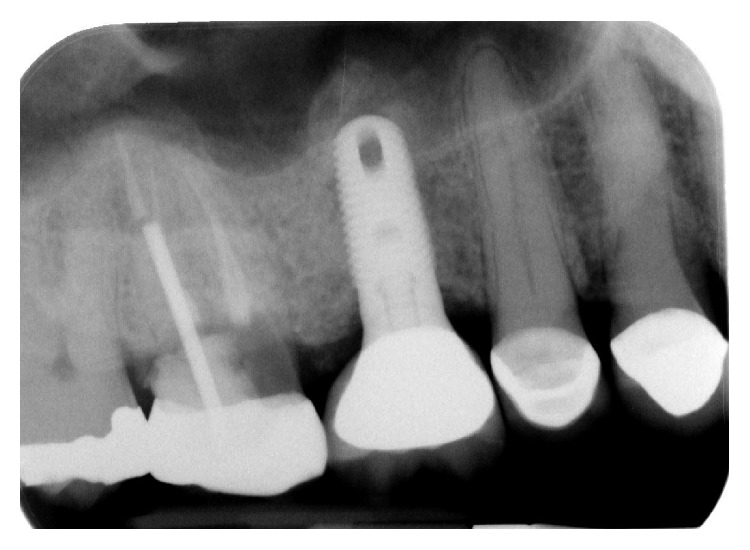
A periapical radiograph was taken after the implant placement. Bone condensation is noted apical to implant #3 due to the use of the osteotome sinus floor elevation technique. Mesial of #2, a deep unrestorable decay was observed.

**Figure 11:**
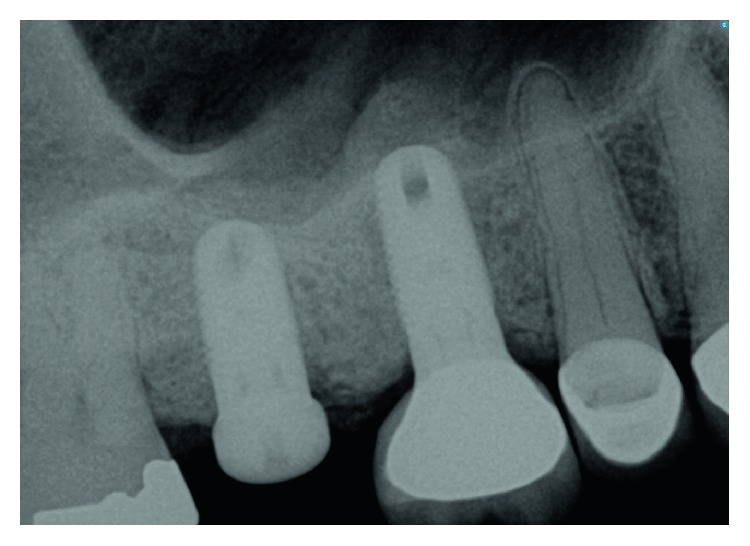
The same protocol of extraction, ridge preservation and subsequent implant placement, was followed successfully for site #2. The implant at site #3 did not show any signs of infection or inflammation, radiographically nor clinically.

The dental history of tooth #3 revealed periapical radiolucency in 2013, on the mesial buccal root (Figure 1), measuring ∼5.4 × 8.7 mm in a cone beam CT (Figure 2). A Seibert Class I ridge deformity was noted at the buccal wall of #3. Upon flap reflection, a fenestration was noted penetrating the buccal wall at the site of the mesiobuccal root apex. The tooth was sectioned, extracted, and a thorough debridement of the socket was performed. Valsava testing was performed to exclude the possibility of communication with the sinus cavity. Freeze-dried bone allograft (FDBA), a collagen membrane, and a nonresorbable high density PTFE membrane were used for ridge preservation and grafting of the buccal plate of area #3 (Figures 3–5). The site was healed by secondary intention. A periapical X-ray was taken with the surgical guide before the implant placement, showing no residual radiographic pathology (Figure 6). The implant osteotomy was prepared with the use of osteotome sinus floor elevation technique [18]. A 4.7 × 11.5 mm Zimmer TSV implant (Zimmer Biomet, Palm Beach Gardens, FL) was placed. The implant was torqued in 35 N/cm, and a healing abutment was placed (Figures 7–9). The implant was referred to the predoctoral clinic for final restoration with cement-retained porcelain fused to metal (PFM) crown (Figure 10). The same surgical and restorative approach was uneventfully followed for site #2, as well (Figure 11). The patient was given an occlusal stabilization splint and was placed on a 6-month maintenance protocol in 2014.

## 3. Case Management

**Figure 12:**
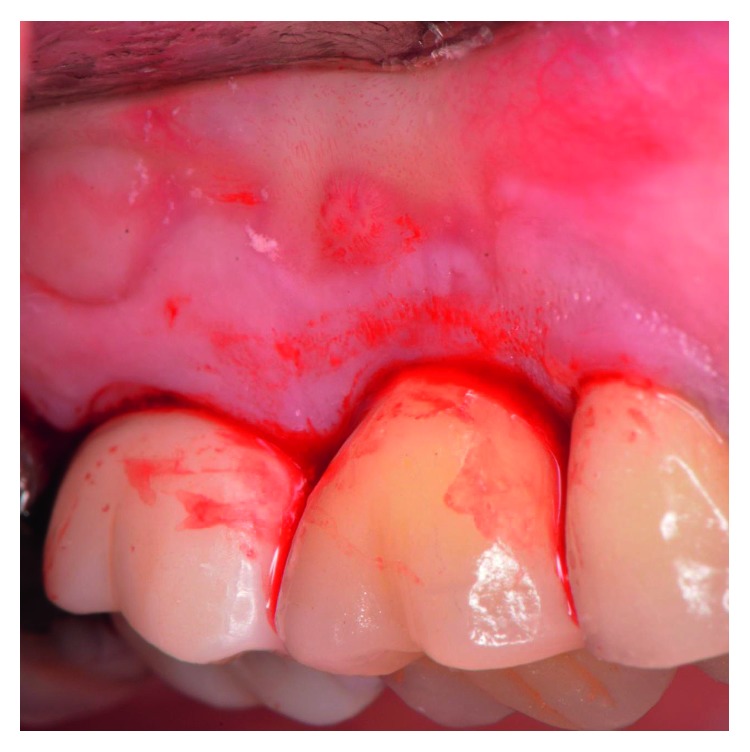
The patient presented at the day of surgical debridement of site #3 with a nondraining fistula at the same site where the fistula was noted before the extraction of #3. The picture is taken after the intrasulcular incisions were performed, with the use of a 15c blade.

**Figure 13:**
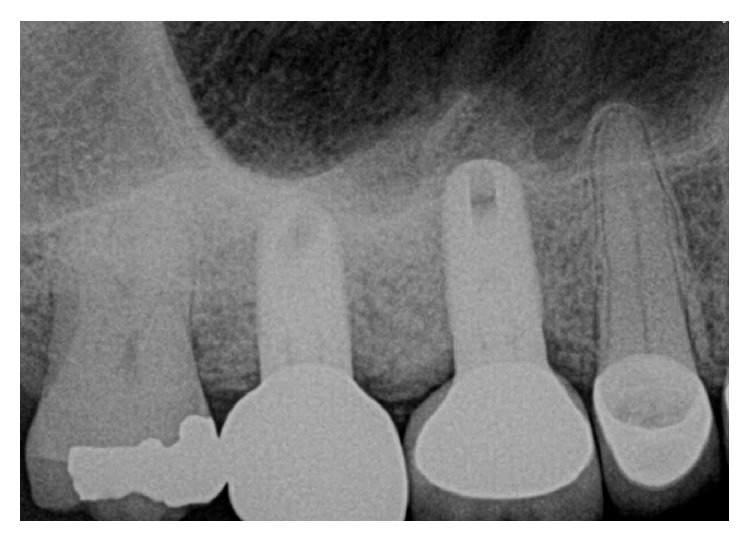
A periapical radiograph on the day of surgical debridement showing a 5 × 5 mm lack of bone density around the implant apex.

**Figure 14:**
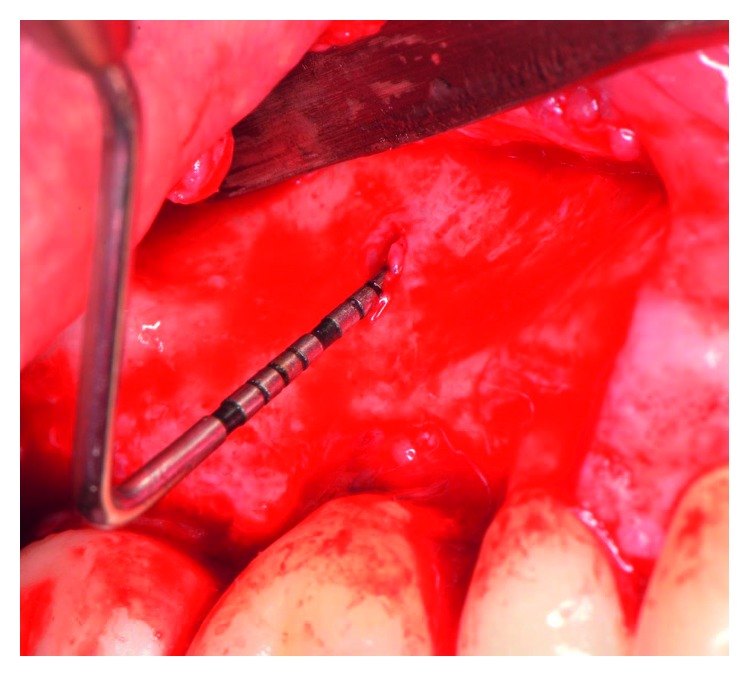
Following reflection of full thickness mucoperiosteal flap, a fenestration of 2 × 2 mm width and 7 mm depth was revealed.

**Figure 15:**
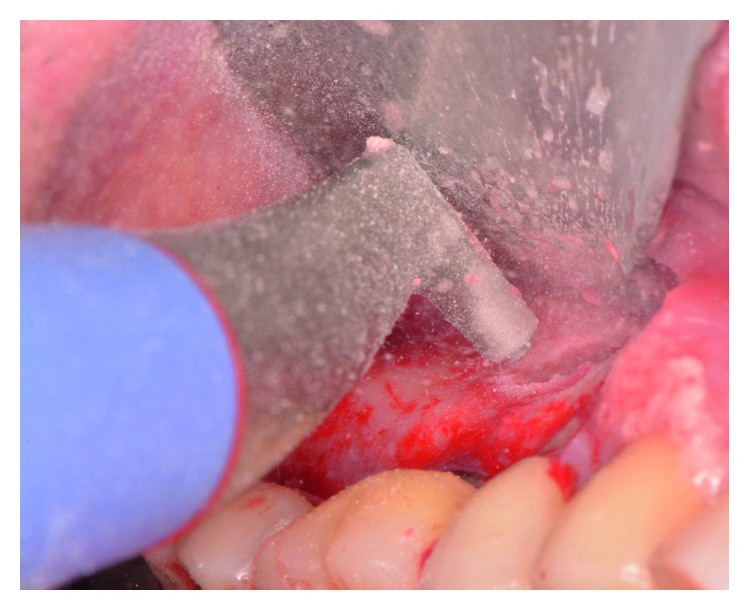
The air powder flow was used to decontaminate the implant surface.

**Figure 16:**
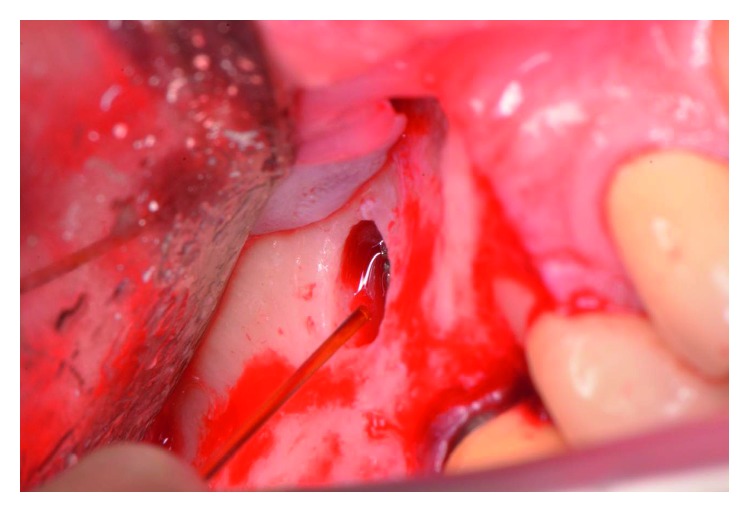
An Er,Cr:YSGG laser with the radially firing periodontal tip used to decontaminate the implant surface.

**Figure 17:**
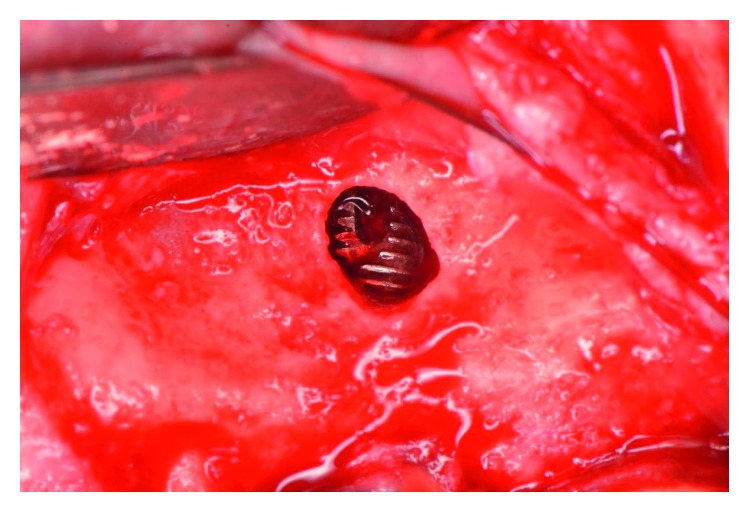
Exposed 7 threads of the implant.

**Figure 18:**
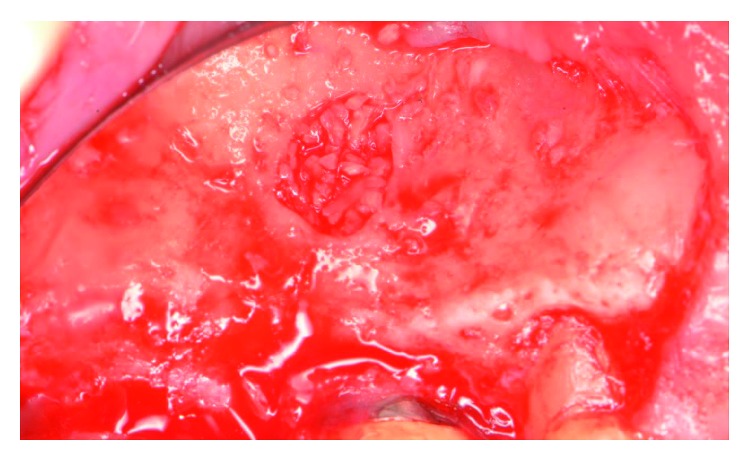
Mineralized cortical bone placed around the apex of the implant.

**Figure 19:**
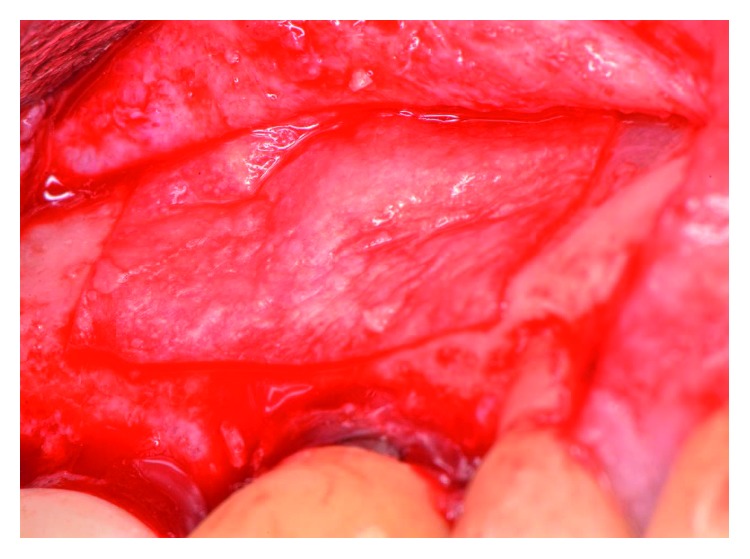
A collagen membrane placed over the grafted site.

**Figure 20:**
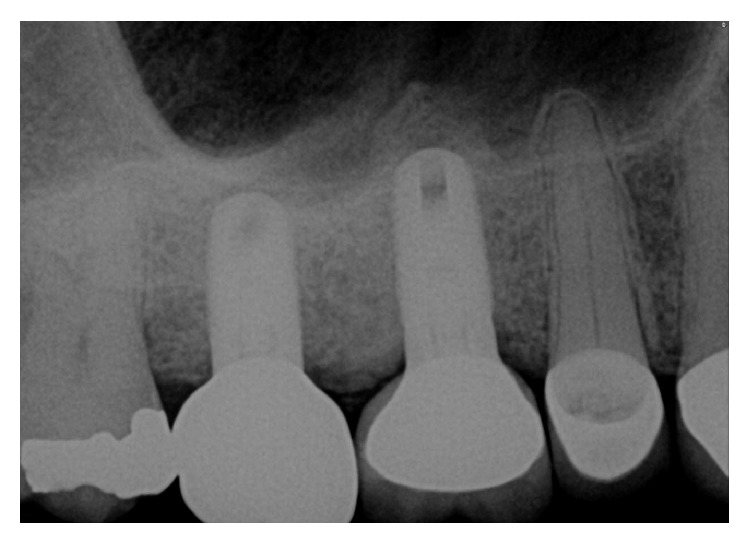
A periapical radiograph at 6 months postoperatively demonstrated an increased density around the implant apex.

**Figure 21:**
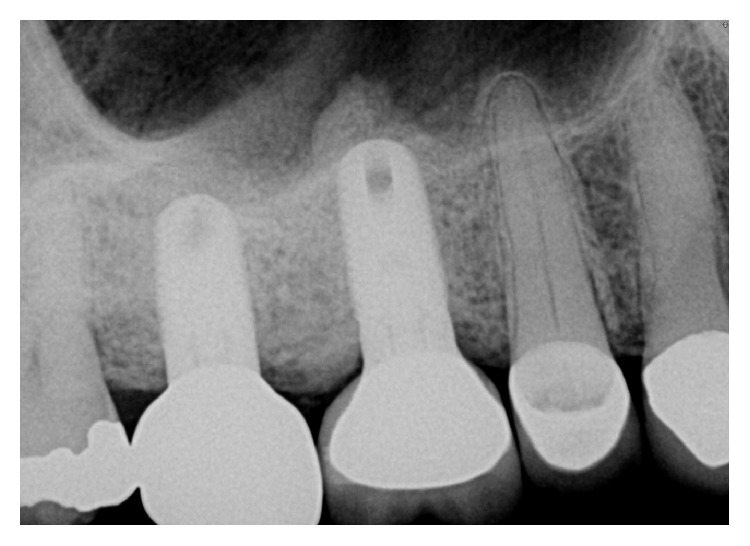
Radiograph at 13 months postoperatively presented increased bone density around the implant apex.

**Figure 22:**
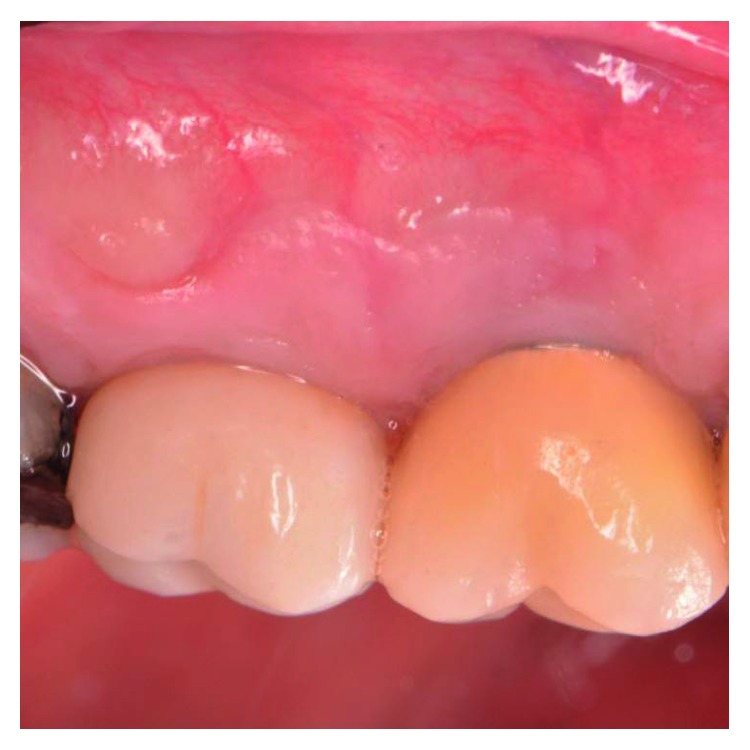
Clinical evaluation 13 months postoperatively showed no signs of pathology.

The patient presented for surgical implant debridement with a nondraining fistula (Figure 12) in July of 2016. The patient understood the benefits and the risks of the surgical approach and signed a consent form. The site #3 implant showed ∼5.5 mm lack of radiographic bone density around the apex (Figure 13). The site was anesthetized by means of local infiltrations, on the buccal and palatal aspects of tooth #3. Intrasulcular incisions extending from #2–4 were performed with a vertical releasing incision placed at the mesial line angle of #4. Upon reflection of a full thickness flap, a fenestration of 2 × 2 mm and 7 mm depth was revealed around the apex of implant #3 (Figure 14). Fibrous granulation tissue was present on the mesial, palatal, and distal aspects of the implant. The bone defect was degranulated using Gracey curettes. No communication with the maxillary sinus was found. The implant surface was initially decontaminated utilizing an air-abrasive device with amino acid glycine powder avoiding direct contact with the implant surface and copious amounts of sterile saline to remove the powder from the implant surface and bone defect (Figure 15). After degranulation, seven threads of the implant were exposed (Figure 16). Before the first laser pass, the patient and the operating staff wore special protective glasses according to U.S. Food and Drug Administration rules [19]. Implant surface decontamination continued utilizing Er,Cr:YSGG laser (with a wavelength of 2,780 nm) at 1.5 W/25 Hz and a radially firing fiber tip (500 *μ*m, RFPT5-14 mm, Biolase Technology, Irvine, CA) (Figure 17). The laser tip was placed perpendicular to the implant surface and 5 mm away from the implant surface. The area was irrigated with saline, and the sequence was repeated (air-abrasive device, sterile saline irrigation, and Er,Cr:YSGG application). Each laser irradiation occurred approximately for 2 minutes. Cortical perforations were performed with a finishing round carbide bur, and FDBA was placed around the implant apex, covered with a collagen membrane (Figures 18 and 19). The flap was repositioned and sutured, without tension, with 4–0 nonabsorbable monofilament sutures. The patient was prescribed amoxicillin 500 mg three times daily for a week, codeine/acetaminophen 30 mg/300 mg every 6 hours as needed for pain management, and a 0.12% chlorhexidine oral rinse twice daily. The postoperative protocol was very strict with biweekly appointments, until the suture removal in 4 weeks. After the initial phase of healing, the patient returned monthly, for the next 12 months in the clinic, for clinical evaluation of the surgical site and radiographic evaluation. The patient healed uneventfully, without any signs of infection or inflammation. Postoperative periapical radiographs from 6 to 13 months showed the increased density of the bone around the implant apex (Figures 20 and 21). The intraoral picture, 13 months postoperatively, displayed no signs of pathology and the probing depths around the implant measured ≤4 mm (Figure 22).

## 4. Discussion

Bacteria associated with failing implants due to infection are similar to those found in chronic periodontitis cases. Therefore, the disruption of the biofilm is a prerequisite for successful treatment [20, 21]. Any peri-implant radiolucency should be addressed immediately to prevent further loss of osseointegration [14].

Reiser and Nevins [4] suggested a classification system for implant periapical lesions differentiating them as either “infected” or “inactive” [22]. The authors suggested a surgical intervention for the infected type and monitoring for the inactive lesion. Recently, a new classification scheme was proposed for RPI with treatment strategies for each class. This classification has 4 classes. Class 1 is when the implant placement results in devitalization of an adjacent previously vital tooth. Class 2 is when an implant apex is infected by a persistent periapical lesion on an adjacent tooth or implant. Class 3 is when an implant apex is placed labial or lingual, outside the alveolar housing. Class 4 is when an implant apex develops a lesion due to residual infection at the placement site. Our case belongs to class 4, which is an implant apical lesion developed due to residual infection. The treatment associated with this class is surgical debridement of the implant site with possible grafting [22]. The management of our case is in accordance with the suggested treatment.

To date, there is no consensus for the treatment of RPI; therefore, the treatment is empiric. Romanos et al., based on a systematic evaluation of clinical case reports, showed that the use of antimicrobials only was not successful in any case for the treatment of implant periapical lesions [12]. The use of an air-abrasive device for the treatment of peri-implantitis compared to mechanical debridement showed significantly better results in BOP reduction after 12 months [23]. Application of air-abrasive powders seems to be an efficacious modality for the decontamination of implant surfaces and is ranking very high, among the other treatment modalities for the removal of the plaque biofilm. Nevertheless, it has an increased risk for emphysema [24]. The preservation of implants' surface integrity is essential even though Ayangco et al. claimed that any scratching during the surgical debridement is not critical [25].

Mohamed et al. presented a case report where a patient was referred for implant placement at the upper lateral incisor (#10) [26]. Both, the central and lateral incisors (#9, 10) had periapical lesions and were treated endodontically. The tooth #10 was extracted due to fracture, and an immediate implant was placed. Four months postoperatively, the implant was diagnosed with RPI due to radiographic and clinical signs of periapical pathology. The implant site was treated surgically with debridement and placement of anorganic bovine bone and platelet-rich fibrin. The authors followed up the case for 12 months. The periapical lesion around the implant apex showed radiographic signs of resolution on the distal aspect, whereas on the mesial aspect, the lesion was still present [26].

Quaranta et al. presented a similar case report of an implant which was placed immediately in a postextraction socket [27]. The extracted premolar (#13) was symptomatic, but further information was not given, nor a radiograph. Three months after the placement, the implant showed both radiographic and clinical signs of RPI. The site was surgically debrided, and a pericardium membrane was placed over the defect without the addition of any grafting material. Five years postoperatively, the implant had no radiographic or clinical signs of residual pathology, and new bone formation was noted around the apex [27].

Ataullah et al. displayed a case where an endodontically treated central incisor (#9) had class III mobility, a post and core and a large periapical lesion [28]. The authors performed extraction and ridge preservation with mixed anorganic bovine bone mineral and autogenous bone. Six months later, an implant was placed successfully on site #9. Two months after the implant placement, the patient presented with a sinus tract between #9 and 10 and a periapical lesion around #9. Implant #9 was diagnosed with RPI, and tooth #10 was vital. The site was surgically debrided and anorganic bovine bone with a collagen membrane was placed. Three months postoperatively, the implant showed no signs of periapical pathology. No further information was given after the first three months postoperatively [28].

Case reports showed that RPI was diagnosed after immediate implant placement in a previously infected area [26, 27]. Therefore, ridge preservation seemed to be a safer approach, when the extracted tooth showed periapical lesions. Nevertheless, like our case, implants were diagnosed with RPI, even if a ridge preservation procedure preceded the implant placement. Implant apicoectomy was suggested in two case reports by Dahlin et al. [29]. Follow-up in both cases showed uneventful healing and absence of clinical symptoms [29]. Quirynen et al. suggested that implant apicoectomy is not required for the treatment of RPI [9]. To achieve complete bone regeneration around peri-implant defects, the use of augmentation materials is required. The concurrent use of GBR following implant decontamination provides stabilization of the blood clot and space maintenance [30, 31]. Implants with periapical lesions that were treated successfully showed a survival rate of 75%, ranging from 4 months to 7 years postoperatively [12].

Different types of lasers are available in surgical dentistry, in various wavelengths, such as carbon dioxide (CO_2_); diode (810–980 nm); neodymium-doped: yttrium, aluminum, and garnet (Nd:YAG); erbium-doped: yttrium, aluminum, and garnet (Er:YAG); and erbium, chromium-doped: yttrium, scandium, gallium, and garnet (Er,Cr:YSGG) [32–34]. During their application, caution is advised not to overheat the implant and therefore compromise the implants' surface integrity. Er,Cr:YSGG laser ablates tissue through a hydrokinetic process and can be used with radially firing periodontal tip and energy settings up to 2.5 W. It does not increase the temperature in critical levels to affect implant surfaces. Furthermore, it successfully removes the plaque biofilm over roughened surfaces, compared to plastic curettes and chlorhexidine [32–34].

Azzeh, showed in a peri-implantitis case report that the use of Er,Cr:YSGG laser enabled the regenerative osseous surgery around an implant. In his case report, the laser was used for flap reflection, as well as for cortical perforations. The results were comparable to our clinical case report, achieving bone regeneration without any complications and with high patient satisfaction [35]. Al-Falaki et al. used Er,Cr:YSGG in a case series of nonsurgical management of peri-implantitis. They treated 28 implants with a mean PD of 6.64 ± 1.48 mm. Six months after the treatment, the PDs were decreased to 2.97 ± 0.7 mm, and the BOP reduction was significantly reduced compared to baseline [36]. Like Er,Cr:YSGG, the use of a CO_2_ laser helps avoid implant surface damage, and the temperature is not increased in critical levels [37, 38]. On the contrary, the use of a Nd:YAG laser could lead to detrimental effects and melting of the implant surface due to overheating since it is being absorbed by the implant surface [39].

Schwarz et al. performed a controlled clinical study comparing Er:YAG versus mechanical debridement with chlorhexidine, in moderate and advanced peri-implantitis cases. The results in terms of reduction of PD and CAL were not significant at 12 months. The most interesting result of this study is that 12 months postoperatively, all patients were discontinued from the study and received further laser treatment and GBR. The reason for that decision was the increased BOP after 12 months of healing [40].

To the best of the authors' knowledge, this is the first case report to describe the concurrent use of an air-abrasive device, Er,Cr:YSGG, and GBR for the treatment of RPI. Implant #3 was placed one year after extraction of tooth #3 and ridge preservation. The implant was placed according to the restorative needs of this site; however, the apex of the implant was positioned in the approximate location of the previous periapical radiolucency of the mesial buccal root of tooth #3. Even though no signs of infection or inflammation were present at the site before and/or after implant placement, RPI was diagnosed 2 years after the implant placement. Dahlin et al. suggested a more aggressive debridement due to the rough surface of the implants. The implants with rough surfaces create an environment where further progression of the RPI or peri-implantitis occurs. Our treatment was in accordance with this suggestion, with the use of an air-abrasive device and Er,Cr:YSGG. The aim of our approach was, due to limited surgical access, to avoid leaving any locus minoris, allowing the bacteria to nevertheless reside in the implant surface after the end of the surgical debridement phase [29]. The subsequent use of GBR was to allow the stabilization of the blood clot and the space maintenance to facilitate the regeneration of the bone around the implant apex. Our results demonstrated radiographic bone fill around the apex of the implant, without radiographic or clinical signs of residual pathology during 13 months of follow-up. Further clinical and radiographic follow-ups are required to provide evidence of this combined surgical approach.

## 5. Conclusions

The etiology of RPI in this case was the placement of an implant in a previously infected site. This case of RPI was treated through a surgical approach utilizing an air-abrasive device, Er,Cr:YSGG, and GBR. The site was monitored for 13 months, and increased radiographic bone density was noted.

## Figures and Tables

**Table 1:** Etiological factors of RPI.

At the time of implant placement	Preexisting disease related to a tooth
(1) Contamination of the surgical bed	(1) Endodontic pathology associated with an extracted tooth
(2) Excessive heat or compression over the time of implant placement	(2) Retained root tip
(3) Presence of remnants of milling	(3) Preexisting bone disease
(4) Overextended osteotomy	(4) Adjacent tooth with periapical radiolucency
(5) Presence of a foreign body	(5) Remaining cells from a cyst or granuloma
(6) Premature loading leading to bone microfractures	
